# Neighborhood Ethnic Diversity, Sense of Community, and Wellbeing Among Native and Migrant Older Adults

**DOI:** 10.1093/geroni/igaf122.2724

**Published:** 2025-12-31

**Authors:** Max Bloem, Jane Cramm, Anna Nieboer

**Affiliations:** Erasmus University Rotterdam, Rotterdam, Zuid-Holland, Netherlands; Erasmus University Rotterdam, Rotterdam, Zuid-Holland, Netherlands; Erasmus University Rotterdam, Rotterdam, Zuid-Holland, Netherlands

## Abstract

Sense of community (SoC) is a key contributor to older adults’ wellbeing, fostering social support, health, and overall wellbeing. As aging-in-place policies and age-friendly community initiatives gain prominence in urban planning, understanding how SoC influences the wellbeing of older adults in diverse neighborhoods is crucial. This study examines how native and migrant older adults from Dutch, Surinamese, Turkish, and Moroccan backgrounds experience neighborhood ethnic diversity in relation to SoC and wellbeing in the Netherlands. Using data from a large-scale study on aging in urban neighborhoods, a regression model was applied to assess the relationships between neighborhood ethnic diversity, SoC, and subjective wellbeing. Ethnic diversity was measured using three indicators: outgroup size, a group-specific Herfindahl-Hirschman Index (HHI), and perceived ingroup size. Findings indicate that ethnic diversity has a limited or no effect on the wellbeing of native-Dutch older adults, while producing mixed effects for migrant groups. Of all the diversity measures, outgroup size had the most significant effect on wellbeing, though not uniformly. It had a positive effect for older adults with a Moroccan background, while a negative effect was observed for Surinamese older adults. This study highlights the importance of considering ethnic diversity in conceptualizations of age-friendly communities, recognizing differences between native and migrant older adults, and acknowledging diversity within migrant groups in research and policy.

